# ‘Getting our voices heard in research: a review of peer researcher’s roles and experiences on a qualitative study of adult safeguarding policy

**DOI:** 10.1186/s40900-022-00403-4

**Published:** 2022-11-28

**Authors:** Lorna Montgomery, Berni Kelly, Ursula Campbell, Gavin Davidson, Leanne Gibson, Louise Hughes, Jadzia Menham, Linda McKendry, Leslie-Anne Newton, Alex Parkinson, Ethan Redmond, Joseph Turnbull, Paul Webb, Lisamarie Wood

**Affiliations:** 1 Queen’s University Belfast, Belfast, BT7 1NN Northern Ireland, UK; 2 Compass Advocacy Network, 20 Seymour St, Ballymoney, BT53 6JR Northern Ireland, UK; 3 Association for Real Change, Wildflower Way, Belfast, BT12 6TA Northern Ireland, UK; 4 Praxis Care, 25-31 Lisburn Road, Belfast, BT9 7AA Northern Ireland, UK

**Keywords:** Learning disability, Adult safeguarding, Adult protection, Co-production, Participatory design, Legislation, Policymaking

## Abstract

**Background:**

Historically, disabled people have been marginalised in research that traditionally adopted a medical model perspective. Since the 1970’s, there has been a shift from research on disabled people to research with disabled people with a strong emphasis on co-produced participatory research. Co-production involves disabled people working with academics to produce research and outcomes which are informed by the end user. This paper reflects on the role and experiences of peer researchers in co-producing a recent UK-wide research project called ‘Getting our Voices Heard’. This project sought to identify the best approaches for people with a learning disability and their supporting organisations to influence adult safeguarding policies, across the four jurisdictions of the UK.

**Methods:**

A co-produced participatory design was used to address the project aims; achieved through the establishment of a collaborative research team comprising academic researchers, key stakeholders and six peer researchers, each of whom had a learning disability. Semi-structured interviews were completed with senior policy makers. Following this, in each of the four Nations, an organisational case-study was completed (four in total). Organisations were purposively sampled to identify one organisation in each country which was recognised as being successful in influencing adult safeguarding policy. Data were gathered through focus groups discussions and semi-structured interviews with key stakeholders. Findings were developed into an Impact Strategy and Action Plan. Co-production methods were used throughout.

**Results:**

Six individuals with a learning disability were recruited and trained to work as peer researchers, involved at key stages of the project, working alongside a wider research team. The role and experiences of the peer researchers in the context of policy are presented. Peer researchers provided largely positive first-hand accounts of their experiences. The importance of collaboration, the invaluable role of Learning Disability support organisations, and the need for additional time and resources to facilitate co-production, was noted.

**Conclusion:**

Whilst peer researchers were positive about their experiences, some success in promoting co-produced research and areas for improvement were evident. Collaboration at all stages would have been strengthened with research funding which enabled involvement of all team members in all research activities.

## Plain English summary

Since the 1970’s, there has been a shift from research on disabled people to research with disabled people. This is often referred to as co-produced research. Co-production has a wide definition but includes disabled people working with academics to produce research and outcomes which neither group could achieve by working in isolation. This paper presents the co-production methodology used to conduct a research project called ‘Getting our Voices Heard’. This project, sought to find the best way to get the voices of people with a learning disability heard inpolicy development in the UK.

We explain how this research was carried out, using a co-produced participatory design. We established a research team with professional researchers from a university, who worked with peer researchers. Peer researchers are people who have lived experience of the issue being studied. In this project, we worked with six peer researchers who all had a learning disability.

The experiences of the peer researchers, and ways in which the peer researchers were involved at each stage of the project are discussed. The peer researchers described feeling positive about their role and felt involved. We show that working together is important and recommend that additional time and resources  are  essential for this joint working.

## Background

Whilst all citizens, regardless of age or disability, are protected by the range of criminal and civil laws that govern their country, many governments have provided additional legal protections for citizens whose personal characteristics or circumstances render them at increased risk of abuse or exploitation. There is considerable variation in this legislation, including variation in how abuse is defined and in the powers and duties of professionals to intervene [26]. Arguably, in this process it is the voice of the professional which predominates in defining adult abuse and shaping the legislative framework in response. This is a critical issue for individuals with a learning disability who experience significant levels of abuse, and are subject to overlapping systems of discrimination and risk [6, 7, 28]. Whilst legislative advances have been made to safeguard such individuals, professional opinion is privileged in shaping government responses to adult safeguarding. This is due in part to the harmful stereotypes and an adverse social construction of disability, and is at odds with the move towards co-production in which disabled people should have a say in every aspect of their life.

The concept of co-production is broadly defined. The term is used generically to describe partnership working between service providers and people who draw on care and support services, to improve those services. Understood in the context of health and social care, Martin [20] outlines two broad rationales for patient and public involvement in health care: the technocratic rationale that prioritises the benefits to the organisation or professional needs,and the democratic rationale that is rights-based and focused on the benefit to service users and redressing inequalities in power or influence between service providers and service users. Williams et al. [33] reflects on the impact of these differing rationales on the implementation of co-production in research, policy and practice and positions co-production squarely within the democratic rationale, defining it as: “a process whereby professionals and those traditionally on the receiving end of their ‘expertise’ (e.g. patients/service users/marginalised citizens) can collaborate with the goal of achieving outcomes that arguably cannot be achieved otherwise. It should engage the talents and experience of all involved…. inherent to coproducing research is bringing together patients/service users/marginalised citizens with researchers and professionals/practitioners and attempting to form equitable partnerships” [33, 3–4]. Co-production, therefore, should involve service users at all stages and utilise an egalitarian, power sharing process.

### Participatory disability research

Historically, disabled people have been marginalised in research that traditionally adopted a medical model perspective focused on individual impairment and ignored the voices and priorities of disabled people themselves [30]. Since the development of the UK social model in the 1970’s, there has been a shift from such research *on* disabled people to research *with* disabled people with a strong emphasis on participatory research that recognises the lived experiences of disabled people and the valuable contribution disabled people can make to disability studies [34].

Whilst this shift to participatory approaches to disability research is welcomed, the danger of tokenism embedded in normative academic-led research and the pervasiveness of power imbalances continue to challenge true co-production [33]. For example, applicants to funding bodies requiring evidence that service users have been involved in the development of proposals can adopt a tick-box approach to one-off consultation with service users rather than engaging in an ongoing collaborative research process where control and leadership of the research agenda is shared [1, 12].

Whilst co-production is advocated within contemporary disability studies, the political and practical challenges of co-production need careful consideration and advance planning in collaboration with disabled people [5, 19]. There are practical and technical barriers that need to be addressed. For example, inflexible institutional payment systems can create barriers to adequate payment for co-researchers’ time and expenses and casual payments can also impact on co-researchers’ welfare benefit income. However, there are also deeper political and attitudinal barriers to co-production that require a shift in normative research processes to allow time to invest in relationships that can sustain co-production and flexible processes that can accommodate an evolving co-production process shaped by all [1]. Chinn and Pelletier [4] also raise the challenge of balancing power relations between non-disabled academics and co-researchers with learning disabilities where not everyone is equally expert/inexpert and the importance of valuing both expert academic knowledge and expert experiential knowledge of co-researchers with learning disabilities based on their lived experience.

Representing the intersectional identities of disabled people is a further challenge drawing attention to how issues such as, age, impairment effects and gender can impact on the co-production of disability research [4, 19]. Co-producing research with disabled people, therefore, requires a critically reflexive approach with careful consideration of accessibility and inclusive approaches [17, 23]. More specifically, people with a learning disability are often considered to lack capacity to engage in all levels of the research process [22]. Chinn and Pelletier [4] also express concern that co-production with people with a learning disability tends to include the same individuals or groups of people who are already experienced in co-production practices or involve more acquiescent individuals who are less likely to challenge institutional research norms. People with a learning disability participating in research may also need access to research training and support to facilitate their involvement (as is the case for any new researcher). Co-researchers should also have choices about how and to what extent they want to be involved in each stage of the research process to utilise the strengths of all members of the research team and respond to the interests of team members [16]. These choices should not be restrictive, and a range of co-research roles should be encouraged and supported to ensure disabled co-researchers are not side-lined to advisory or data collection roles but are also enabled to participate in the conceptualisation of research questions, the design of methods and the interpretation and write up of findings.

Atkin et al. [1] argue that a fundamental basis for co-production is an inclusive, collaborative process based on shared power, flexibility, trust and willingness to engage in mutual learning. Co-produced disability research requires a firm commitment to working alongside disabled people and opportunities for disabled people to shape the research, critique approaches and contribute ideas. People with learning disability have been involved in the identification of research questions [9], data collection and analysis [18, 31], reflection [2], and dissemination [10].

At the core of co-production is an openness to different perspectives that may challenge personal or professional assumptions and a willingness to compromise and share ownership of the research, working towards new-shared knowledge [1]. Such collaborative processes inevitably require additional cost, time, effort and emotional labour from all involved, but ultimately lead to a more egalitarian and reciprocal and rewarding research process [33]. Indeed, it is clear from the experience of those who have engaged in co-produced disability research, that the benefits have far outweighed the challenges [5, 16, 19].

The implementation of a genuine co-produced project requires careful consideration, and it is therefore intended that this paper will contribute to the body of literature that explores the process of co-production with people with a learning disability, including both the strengths and the challenges. An overview of the co-production methods will be presented in relation to a recent UK-wide research project called ‘Getting our Voices Heard’ (GOVH). GOVH sought to identify the best approaches for people with a learning disability and their supporting organisations to influence adult safeguarding, and associated policies, across the four jurisdictions of the UK. Whilst there is a wide body of literature exploring the contribution of disabled people to disability studies [34], people with a learning disability are often under-represented in policy making processes in general and even when the focus is disability [13]. This paper will add to the literature by exploring the co-production of policy related research.

## Methods

### Project infrastructure and local context.

This project, was funded by Disability Research on Independent Living and Learning (DRILL), and was part of a wider DRILL research programme seeking to build better evidence about approaches which can enable disabled people to achieve independent living, and, as full citizens, take part socially, economically and politically within their societies. In this context, DRILL commissioned research on approaches that disabled persons have taken to exerting influence on a specific policy area.

The original idea for the project was identified by individuals with a learning disability involved in a self-advocacy group, called Telling It Like It Is (TILII). TILII is a group of over 50 self-advocating adults with a learning disability who live within community and hospital settings in Northern Ireland (NI), and who were experienced in voicing their views regarding policy and practice. They were concerned about their lack of input into safeguarding procedures, suggesting that the current adult safeguarding policy in NI did not meet their needs. They gave examples of responses to safeguarding incidents in which they perceived either that the safeguarding issue was not being taken seriously or the response was too invasive. They initiated a process which engaged both academics and learning disability support organisations to develop a research brief and grant application. This collaboration was eventually successful in securing funding through the DRILL programme, to carry out this research.

Following the award of the DRILL grant, a research infrastructure was established which sought to be inclusive of all sectors involved in the project. The project structure had four main components: the DRILL Programme itself, which funded the project and provided oversight, monitoring and accountability; secondly, an Advisory Group, whose membership included six strategic safeguarding stakeholders from across the UK, including academics, and two individuals with a learning disability. The Advisory Group  had four meetings with the research team during the lifecycle of the project, offering strategic oversight and consultation. Thirdly, TILLI took on the role of the Peer Reference Group and ensured their involvement in the development of the methodological approach and project plan. They provided a mechanism for piloting research tools and assisting with the development of accessible and easy read versions of any produced materials. Finally the Project Team itself included: six PRs with learning disabilities, based in either the Association for Real Change, Northern Ireland (NI) or Compass Advocacy Network (CAN), NI; a senior staff member from ARC and CAN and Praxis Care, three academics from Queen’s University Belfast, and a research  officer from Praxis Care. The principle author (LM) has a research and practice background in adult safeguarding. All research team members had prior training in understanding adult safeguarding. All team members are authors in this paper.

The Association for Real Change (ARC) is one of the leading organisations in the learning disability sector operating across the UK, with a lobbying role and training function. Compass Advocacy Network (CAN) is a Disabled Person’s Organisation (DPO) (an organisation which is run and controlled by disabled people), operating in NI, empowering people with a learning disability to self-advocate on issues such as sexual health, welfare reform and community planning. Praxis Care is a voluntary sector organisation which support people with mental ill health and learning disability. The ARC and CAN representatives were members of the core research team, with an essential role in facilitating the co-production of the project.

A co-produced participatory design was used to address the following aims of the GOVH project, with the full project methodology and findings published in a separate article [24].

- Identify the different approaches to exerting influence on adult safeguarding policy that have been taken by people who have a learning disability and their support organisations;
- Explore what works in different contexts by looking closely at successful examples where people with a learning disability and support organisations have influenced adult safeguarding policy or practice; and
- Make recommendations on approaches to take and identify barriers and enablers to exerting influence on future adult safeguarding policy and legislation.

The project was divided into two key stages. Stage one primarily focused on data gathering and stage two on promoting impact. Research methods are discussed here largely in terms of the role and experiences of PR; outlined in Table 1.

**Table 1:** Research team roles and responsibilities

Research activity	PR	Research assistant	Academic researcher	Learning Disability organisations	Support worker	Timeline
Recruitment of PR	Prepare and attend for interview (8 individuals interviewed)	Member of interview panel	Member of interview panel	Chair interview panel	Support applicant during interview	March 2019
Training of PR	6 PR selected and attended training	Prepare and deliver training	Prepare and deliver training	Undertake training needs analysis with PR Prepare and deliver training	Support PR during training	4 half days April 2019
Semi-structured interviews with 8 policy makers	Co-produce interview schedule Co-facilitate interviews: 4 PR undertook 1 interview each 2 PR undertook 2 interviews each	Co-produced interview schedule Co-facilitated interviews with PR	Recruit study participants Debrief interviews	Debrief interviews	Support PR in interviews	March-June 2019 See Table 2
Focus group discussions and semi-structured interviews within 4 ‘best’-case organisations	Co-produced interview/FGD schedules Travelled to each organisation. PR co-facilitated in 1 or 2 case studies each. See Table 4	Co-produced interview schedule Travelled to meet members of each organisation Co-facilitated in all research interviews and FGD See Table 4	Debrief interviews and feedback on process	Debrief interviews and feedback on process	Travelled with PR to support them during data gathering	September 2019: Edinburgh September 2019 Belfast October 2019 Cardiff October 2019 London
Impact Strategy/Action plan	Co-developed impact strategy/action plan Presented at one Action Planning workshop Video made for remaining workshops	Co-developed impact strategy/action plan Presented at one Action Planning workshop	Co-developed impact strategy/action plan Presented at workshop in person/online	Co-developed impact strategy/action plan Presented at workshop in person/online	Support PR during workshop and to read and respond to all draft documents	March 2020

### Stage one

#### Recruitment and training of peer researchers

Six PRs, individuals with a learning disability, were recruited from ARC NI and CAN using a structured recruitment process involving a formal application and interview. The posts were advertised through ARC and CAN using an easy read information leaflet. Nine individuals applied for these posts, with eight offered an interview. Interviews sought to assess the core skills needed to act as a PR, namely that individuals’ required good communication skills, an ability to understand the nature of the project and to understand the role of a PR. Support was offered to potential applications to prepare for interview by a support worker from the organisations with which they were associated. Unsuccessful applicants were invited to meet the panel Chair for feedback. Once selected, the PR committed to contribute to weekly involvement, over the year-long process. As institutional payment and welfare benefit systems created barriers to adequate payment of PRs, payment was organised through a voucher scheme. The three male and three female PRs ranged in age from 22 to 60 years, and PRs had been diagnosed with moderate learning disabilities. Each PR was living in the community at the time of the research, had engaged adult social care services and currently had additional daytime activities or part-time work.

PRs were provided with an induction to the project and four half-day formal training sessions (see Table 2).The training curriculum was based on a training needs analysis conducted by the research team, and was also informed by an experienced PR from a different project who contributed to the planning and delivery of the training. As part of the training, PRs undertook a formatively assessed role-play of a mock interview with a ‘policy maker’, with written feedback given on their performance.

**Table 2:** Curriculum for peer researcher training

Day 1	Introductions and working together Overview of research purpose and process Overview of adult safeguarding
Day 2	Core research skills: How to introduce yourself Explaining the project to others How to begin to ask questions Developing Questions for the project
Day 3	An overview of how laws and policies get made Working on research scenarios and dealing with difficulties Ethical dilemmas: Experiential Workshop: Role Play opportunities (1)
Day 4	Managing Self disclosure and boundaries Promoting Self care Exercise—Wellness Recovery Action Plan Experiential Workshop: Role Play opportunities assessed (2). Evaluation of each role-play with written feedback on strengths and areas for development for each PR

#### Semi-structured interviews with policy makers

Semi-structured interviews were completed with an identified senior policy maker or civil servant and/or politician in each of the four UK Nations, namely: England, Scotland, Wales and NI ( see Table 3). The interviews were conducted either in person or using an online platform or telephone call.

**Table 3:** Policymaker interviews

Region (UK)	Number of interviewees	Occupational area
Northern Ireland	4	Policy makers Health and social care leads
Scotland	1	Policy maker
England	1	Policy maker
Wales	2	Politicians

The PR role involved developing interview schedules in consultation with the other members of the research team and the Peer Reference Group. These interview questions were based on a desk based analysis of relevant literature and policy, which was undertaken by the RA, and is not reported here. In so doing the PRs together with the other research team members, reviewed broad topics for interview and developed these into a series of questions. Questions focused on the development of policy, specifically exploring ways in which policy is influenced by learning disability support organisations. Easy read versions of the questions were developed to assist the PRs. The six PR were supported to prepare a script around introducing themselves and asking three key questions, as a base-line. Four PRs also prepared a script to introduce the project, engage more fully and responding to answers. During this process, and throughout the project, the support worker met with the PRs to assist them to understand and respond to written material such as draft research interview questions.

PRs jointly conducted the interviews along with the research assistant (RA). The support worker was available to attend the interview if needed, and for example, rephrased any dialogue they did not understand. The level of support and shared input was decided in advance with a written plan agreed for each interview and prompts agreed to manage particular scenarios. The interviews were audio-recorded (with consent), transcribed and co-analysed. Interviews lasted between 20 and 60 min.

#### Organisational case-studies

The use of exemplar case studies of organisations that have been successful in policy influencing has been identified as an effective approach to learning in this area [21]. In each of the four Nations, an organisational case-study was completed (four in total). Organisations were purposively sampled to identify one organisation in each country which was recognised as being successful in influencing adult safeguarding and associated policy. Data were gathered through focus groups discussions (FGD) and semi-structured interviews with key stakeholders in each organisation, including staff, family carers and individuals with a learning disability (See Table 4). Questions focused on ways in which the organisation had co-produced their influencing strategy, and ways in which the organisation sought to influence safeguarding policy and its implementation at national and organisational levels. The impact of devolution and differing policy contexts and structures across the UK was also considered. Where individuals with a learning disability were participants, the communication needs and preferences of participants were shared prior to interviews. Easy read versions of the information sheet and consent form were developed in partnership with the PRs.

**Table 4:** Organisational case studies

Region (UK)	Case-studies	Organisation
Belfast, Northern Ireland September 2019	Interviews with 2 senior staff: members and 2 learning disabled members 1 FGD comprising staff and members	Disabled Person’s organisation (i.e. Majority of the Board members have a learning disability)
Edinburgh, Scotland September 2019	Interviews with 2 senior staff: members and 2 learning disabled members 1 FGD comprising staff and members	Disabled Persons organisation
London, England October, 2019	Interviews with 2 senior staff: members and 2 family carers 1 FGD comprising staff and carers	Disability Support Organisation
Cardiff, Wales October 2019	Interviews with 2 senior staff: members and 2 learning disabled members 1 FGD comprising staff and members	National member-led organisation for self-advocacy groups and people with learning disabilities

In facilitating these organisational case-studies, four research sub-teams were established from within the project team, each responsible for one of the four UK nations. A sub-team consisted of two or three PRs, the academic researcher and the support worker. The sub-teams travelled across the UK to complete data collection in each organisation. Again, the interview and FGD schedules were drafted in consultation with the PR and the Peer Reference Group. Semi-structured interviews were conducted jointly by one PR and the RA. FGD were conducted jointly by two PRs and the RA. A plan was agreed as to how to balance these roles. All interviews and FGD were audio-recorded (with consent), transcribed and co-analysed. Interviews lasted between 35 and 70 min. FGD lasted between 45 and 70 min.

Following this data collection, an online survey was sent to support organisations who contributed to safeguarding policy consultations (identified in the policy analysis of this project), and disseminated through the ARC UK network. This process did not involve PR, and is not reported here.

### Stage two

A co-produced Impact Strategy/Action Plan was developed based on the evidence and analysis from Stage one, seeking to influence adult safeguarding policy at regional levels across the UK. It included plans for dissemination of the key messages from the study to, for example, Disabled Persons Organisations, policymakers, academics, and statutory bodies such as Adult Safeguarding Boards. The first draft of key themes was shared with the PRs and with the Peer Reference Group for iterative development. The Advisory Group, were also consulted. This plan included key recommendations on co-producing policy development for both policy makers, and disabled people and their supporting organisations.

### Data analysis

Research data were audio recorded and fully manually transcribed by the RA; transcripts were then analysed by the RA and one academic researcher. Data analysis took place in the form of exploratory applied thematic analysis, drawn from the work of Guest et al. [11], with the help of NVivo software in order to help organise the data in an accessible manner. Individual interviews and case studies, were analysed separately. Drafts of key themes  were shared with the PRs and with the Peer Reference Group for iterative development in order to enhance both the accuracy and usefulness of the findings.

### Exploring peer researcher’s roles and experiences

The views of PRs were gathered through qualitative methods, these included debriefing opportunities throughout the project along with qualitative interviews at its conclusion. Further reflections on the co-production processes were provided by the wider team members at each of the monthly team meetings and at a specific review of the co-production process towards the end of the project.

### Ethics

The project was given ethical approval from the Research Ethics Committee in the School of Social Sciences, Education and Social Work, Queen's University Belfast. The key ethical considerations included ensuring that participation was voluntary, that confidentiality was protected, and that people were given support, when needed. It was acknowledged that training needs should be addressed at the start of the project and that training would include guidance on how to respond to ethical or safeguarding issues which might arise. This included the inclusion of a distress protocol and a safeguarding protocol.

## Results

### Recruitment and training: PR experiences

The PRs identified their motivation to apply for this role as relating to both personal development and a commitment to the values of the project. One PR described his motivation thus, *‘It is important that I make life better for others like myself and this job is going to make sure policy makers did not forget people with a learning disability’.*

Reflecting on the recruitment process, PRs were positive about the process and the level of support given to them. For example, one PR stated, *‘I felt the interview process was positive. I was able to fill in the application with help and was encouraged to share strengths and experience*. This helped me to get the interview’. Another PR commented on how nervous he felt at the interview. ‘*I felt nervous but could see that everyone wanted me to do my best. Having all the support was important; it allowed me to feel more confident and enabled me to show my abilities and previous experience’.*

Another PR said he enjoyed the interview process as he practised with a support worker, and had reviewed some of the important things he had done through his life to pick out what would help him to get the job as peer researcher. This PR said he could see how interested everyone was and how pleased they were he had gone for the interview. ‘*When I got the job, I felt fantastic’*.

Training was delivered by members of the research team who had all experience in delivering training to individuals with a learning disability, along with an experienced PR who had worked on a previous project. Formal evaluation of training highlighted the usefulness of the topics being covered, with the benefit of formative assessed role-plays emphasised. The training was also critiqued as creating a sense of belonging, excitement and pride in being involved. The contribution to training sessions of a PR was also viewed as helpful. These positive experiences have been conveyed by the PRs, ‘*The training helped me by giving me the chance to do role-plays to prepare me for the different types of interviews I would be doing’.* Another PR stated that *it ‘was a great opportunity to meet new people, learn new things and be part of an important project’*.

However, challenges to the training were also identified. Formal evaluation highlighted that more experiential methods would be welcome and that four half days were too intense and in parts, needed to be paced differently. Additionally, it was at times difficult to assess if the training was appropriately targeted to the ability of the participants, who had differing levels of capacity. In this context, capacity refers to the general sense of having the required skills and knowledge to undertake the task. It was also acknowledged that top-up training during the course of the project would have been welcome. One PR stated ‘*The training was hard for the two days, a lot to take in and learn. The project took a long time so I had forgotten some of the information and training’.*

### Data gathering stages: PR role and experiences.

As noted, there were two main qualitative data-gathering stages: individual interviews with policymakers, and organisational case-studies. PRs reflected positively on the process, *‘I think everyone took me seriously’* another PR stated *‘It was great to interview policy makers, this felt important. I hope the results make a difference’.*

Whilst the PR acknowledged the stress involved in travelling to England, Wales and Scotland to undertake research with selected organisations, their feedback was largely positive. *‘I enjoyed going to London and speaking with parents and carers at X organisation’.*

PRs were centrally involved in each data gathering stage. In addition to asking questions, it was noted that they were able to show empathy to the family members and individuals who had learning disability during research interviews.

In analysing the data, the RA and one academic identified key themes through a process of thematic analysis [11]; these were shared in draft with the PR and Peer Reference Group for consideration if themes accurately represented the findings, addressed the research questions and were understandable. The Advisory Group also reviewed and commented on the draft findings. The processes identified here ensured the development of interview questions and research processes were informed by the real-life experiences of people with a learning disability.

### Dissemination and impact: the role of peer researchers

A comprehensive project report, an executive summary and a 10-point Action Plan for policy makers and one for learning disabled individuals and their support organisations was co-produced in both standard and easy read formats [25]. In co-producing these reports, a first draft of each was written by the academic researcher and then shared for iterative development with all members of the wider team, including the Peer Reference Group and PRs.

Finally, comprehensive dissemination plans were discussed and agreed by the wider team members with a range of in-person and online dissemination events organised throughout the UK. However, these plans were significantly disrupted due to implementation of Covid-related restrictions. The first dissemination event in NI went ahead with the PRs leading on the presentation of the findings to an audience including policy makers, civil servants and key stakeholders. To compensate for limited travel opportunities, the PRs made a short video of recommendations which were shown at subsequent online dissemination events.

## Discussion

This paper reports on the co-produced participatory design used in the GOVH project. The main aim of the project was to consider the process and impact of the involvement of disabled people in the development of adult safeguarding policy. The main findings from that aspect of the project included the identification of factors which enabled or restrained disabled people, and their organisations, informing and influencing policy and these are reported in a separate article [24].

Another aspect of the project, which is the focus of this article, was to consider the role and experiences of the involvement of people with a learning disability, as PRs, on policy-related research. In conducting this research, an infrastructure was established which sought to be inclusive of different sectors involved in the project, with the establishment of a project research team made up of six PRs with learning disabilities, key representatives of disability support organisations and academic researchers. Findings are presented in terms of the role and experiences of PR at each stage in the project. To a large extent, PR perceived their experiences positively: feeling valued, involved and pleased to have contributed to something that they believed was important. Overall, whilst genuine collaboration and shared ownership was evident, areas for improvement were identified.

### Co-production: early stages of the project

Increasingly people with a learning disability have acted as PRs in studies which have relevance for matters which are important to them [3, 8, 14, 32]. From the outset, the research team were conscious of the risk of adopting a tokenistic approach to co-production. This is often a risk at the stage where projects are conceptualised, for example, where applicants to funding bodies adopt a tokenistic approach to the required involvement of service users [1, 12]. In this project, it was the adults with a learning disability, as members of a self-advocacy group who had identified the need for research in this area, and had engaged key stakeholders. This resulted in a plan, agreed by academics and disabled persons organisations, to seek funding for this research. It was hoped that, if successful, academics would take a secondary role in this process and people with a learning disability and their supporting organisations would assume leadership. However, as identified by Atkin et al. [1], pragmatic barriers exist for many voluntary sector organisations in responding to research funding calls, with prohibitive requirements around insurance policies and financial resources. Largely because of these issues in this project, of the agencies interested in leading the project, only the academic institution had the capacity to act as lead applicant for the funding call. This was potentially disempowering for partners, establishing a power dynamic from the outset with one of the academic researchers acting as Principal Investigator. In seeking to manage this dynamic, a deliberate commitment was made by all team members, to establish systems in which both academic knowledge and expert experiential knowledge was valued [4]. In so doing, the establishment of the Advisory Board and a Peer Reference Group helped to ensure that decisions were shared across multiple sectors and had input from a variety of positions. Monthly meetings, with co-production as a standing agenda item, helped to ensure the distribution of power. However, the potential for tension between the ethos of equality and power sharing and the limits of time and funding to support PR to get involved in each stage of the research process was acknowledged. On reflection, a written ‘terms of engagement’ relating to co-production processes which has been utilised elsewhere [29], would have been beneficial in formally sharing power and ensuring accountability within the participatory design.

### Co-production: recruitment and training stages of the project

Garcia Eriarte et al. [8] argue that caution should be used in adopting an uncritical endorsement of the inherent benefits of inclusive approaches, with issues around recruitment, resources, support and follow-up for PRs identified [8]. In managing the PR recruitment processes careful consideration was given to issues around accessibility and inclusion [17, 23]. As PR posts were advertised through voluntary sector organisations, this limited potential applicants to groups of people who may already have opportunity and experience in co-production practices [4]. An open recruitment process would have been preferable but was not possible within the allocated budget and timescale. Whilst transferrable experience relevant to the role description was valued, most PRs had no prior research experience. PRs acknowledged the importance of support to prepare for interview, which they identified as essential in increasing their confidence and skill to engage in the interview process.

A collaborative approach was taken to the development and facilitation of the training programme, with contributions from a PR from another project. As noted, the PRs evaluated the training highly, with experiential methods favoured. However, PR feedback was mixed; whilst some PR valued the training for others the four half-days were too intense, with training delivered later in the project suggested. The assessed role-play research interviews were positively evaluated by all team members, helping to identify PRs' strengths and support needs. It was informative, particularly for academic researchers, to observe first-hand the strengths of each PR and to see the degree to which PRs could take the lead in research interviews. Arguably, from this early stage, a partnership approach was fostered by collaboratively negotiating a role for each PR which reflected an accurate assessment of their preferences and strengths. Without the formative role-play assessment, it is likely that PRs would have had a more limited role in conducting interviews and potentially a more paternalistic approach would have been adopted.

### Co-production: data gathering, analysis and dissemination

Genuine co-production requires sharing project ownership, an investment in relationship, and a flexible approach through which PRs should be engaged throughout the various stages of the project [1, 12, 33]. Garcia Eriarte et al. [8] suggest that the success of co-produced research is influenced by the interest and persistence of PRs. In this project, to a large extent, the PRs remained engaged throughout the year-long process, a team approach was adopted, with monthly formal team meetings and frequent informal discussions between all team members. PRs were engaged in the majority of research stages and the co-ordination of the project was a shared process. However, in spite of these deliberate efforts to promote genuine co-production, a number of issues were identified. A flexible approach was required for data collection, with the need to plan the specific role that a PR would take in each interview and FGD. This required regular discussions between team members, who arguably became more attuned to PR strengths and encouraged increasing input from the PRs, over time. Secondly, there was a need to clarify the role of the support worker in interviews and FGD. Whilst it was agreed that the support worker should not ask research questions, working out the boundaries to this support required careful negotiation  and reflection after each interview.

Often people with a learning disability are considered to lack capacity to engage in all levels of the research process [27]. In this study, the PRs did not have a role in developing the online survey or desk based analysis of relevant literature and policy. This decision was based on the need to play to the strengths and interests of each team member and the literature review was best suited to the RA who could quickly summarise policy and processes, whilst consultation with the Peer Reference Group was ongoing. However, it is evident that the input of the PRs into these areas would have been valuable, promoting a stronger co-produced study. It is recommended that time and resources should be made available in funding to enable PRs to develop their skills in these areas.

Feedback from the PRs indicated that they felt that they were ‘taken seriously’, and their input valued. This was aided by the ARC and CAN team members who promoted significant involvement of the PRs at each stage of the project. ARC and CAN encouraged a mind-set from all team members to share control over the research in a meaningful way, and an awareness that to do so required additional time to complete data collection, data analysis and dissemination. Whilst, collaborative processes inevitably require additional cost, time, effort and commitment from all involved, they ultimately lead to a more egalitarian, reciprocal and rewarding research process [33].

Finally, the impact of co-production was evident throughout the research process, initially through the partner organisations and then also directly through the work of the six peer researchers. However, the work of the peer researchers may also have had a wider impact by demonstrating and promoting the inclusion of peer researchers in policy related research. People with a learning disability are often under-represented in policy making processes in general, and are underrepresented even when the focus is disability [13]. Although the extent of this impact is harder to gauge, policy makers saw first-hand the strengths of co-production in policy research and were engaged by people with a learning disability on policy considerations. Whilst not accessed as part of this study, policy maker attitudes to disability and the associated stigma which is seen as a barrier to policy influence may have been challenged by the role that PRs took in this project.

## Conclusions

Co-production has arguably become a cornerstone of disability studies that involves disabled people in all stages of the research process from design to data collection, data analysis and impact activities [15]. As noted, Atkin et al. [1] argue that a fundamental basis for co-production is an inclusive, collaborative process based on shared power, flexibility, trust and willingness to engage in mutual learning.

Whilst the PRs in this study provided largely positive first-hand accounts of their experiences, feeling valued, involved and pleased to have contributed to something that they believed was important, this article has identified some success in promoting genuine co-production and areas for improvement. Positively, the invaluable role of the Learning Disability support organisations (ARC and CAN), who had a remit to promote co-production, was identified. The project would not have proceeded without this collaborative approach and commitment to valuing highly their expertise, whilst prioritising the development of team relationships. However, co-production could have been enhanced with a revised PR training schedule, earlier clarity around the PR input into interviews and FGD, and clarity around the role of the support worker. Additionally, collaboration at all stages would have been strengthened with research funding which enabled involvement of all team members in all research activities.

Arguably this research design increased the learning and capacity of everyone involved in the project [15]. In improving the capacity of PRs to engage in research, it is vital that consideration is given as to how PR can be empowered to utilise their research knowledge and skills to engage in future projects.

## Data Availability

Not applicable.

## Abbreviations

ARC: Association for real change
CAN: Compass advocacy network
DPO: Disabled Person’s Organisation
DRILL: Disability research on independent living and learning
NI: Northern Ireland
PR: Peer researchers
UK: United Kingdom
